# Impact of old environmental burden in the Spiš region (Slovakia) on soil and home-grown vegetable contamination, and health effects of heavy metals

**DOI:** 10.1038/s41598-022-20847-8

**Published:** 2022-09-30

**Authors:** Janette Musilová, Hana Franková, Judita Lidiková, Juraj Chlpík, Alena Vollmannová, Július Árvay, Ľuboš Harangozo, Jana Urminská, Tomáš Tóth

**Affiliations:** 1 Institute of Food Sciences, Faculty of Biotechnology and Food Sciences, SUA Nitra, Tr. A. Hlinku 2, 949 76 Nitra, Slovakia; 2 Institute of Agronomic Sciences, Faculty of Agrobiology and Food Resources, SUA Nitra, Tr. A. Hlinku 2, 949 76 Nitra, Slovakia

**Keywords:** Element cycles, Environmental sciences, Risk factors, Geochemistry

## Abstract

Due to several centuries of ongoing mining activities, Middle Spiš (Slovakia) is one of the areas with a damaged environment. The contents of Fe, Mn, Zn, Cu, Ni, Pb, Cd, and Hg were determined in the soils and home-grown vegetables (potatoes, carrots, tomatoes). Except for Pb, the contents of heavy metals in the soils of some plots were higher than the limit values. Based on the values of Contamination factor (C_f_), Degree of contamination (C_deg_), Geo-accumulation index (I_geo_), and Pollution load index (PLI), very high Fe, Cd, and Hg contamination (C_f_ ≥ 6), very high soil contamination (C_deg_ ≥ 20), extremely heavy Fe and Hg contamination (I_geo_ > 5), resp. moderately pollution to non-pollution (1 < PLI ≤ 2) was found in all plots. In vegetable samples, the maximum levels were exceeded for Cu, Pb, Hg (potato), Pb (carrot, tomato), and Hg (carrot, plot E). Bioaccumulation factor values BAF > 1 were for Cu (carrots, potatoes). Estimated daily intake values for all heavy metals were lower than their tolerable daily intake. Chronic daily intake of heavy metals ranged 2.495E−06 (Hg)—0.1416 (Fe) mg/kg/day. Based on Hazard index values, potato consumption poses a risk (0.8068–1.3057). The results showed that the monitoring of soils and cultivated production is necessary for the investigated area.

## Introduction

Environmental pollution is a global problem closely linked to the content of toxic chemicals in the environment^1^. The mining and subsequent processing of complex iron and copper ores in Slovakia have a negative effect on the area of Middle Spiš. The most important mining sites include Krompachy, Spišská Nová Ves, Mlynky, Novoveská Huta, Rudňany, Poráč, and Slovinky. Exceedances of the limit values of Hg, Cu, Zn, As, Cd, and Pb were found in the soils^2^.

Heavy metals (HMs) can accumulate in the edible parts of plants, enter the food chain, and cause adverse toxicological effects to consumers^3,4^. Their accumulation in vital organs such as the liver, kidneys, and bones can lead to many serious health disorders^5^.

Iron (Fe), as an essential mineral, plays an important role in basic biological processes. Fe is also a cofactor of many enzymes involved in the photosynthesis of plant hormones^6^. Although iron (Fe) is essential for most life forms and is widely used in various proteins to perform many functions^7^, there is a presumption that the interaction of iron and cholesterol is a crucial mechanism in promoting oxidative damage that causes atherosclerosis and neurodegeneration^8^.

Manganese (Mn), the most abundant trace element, is closely related to Fe. In plants, it is involved in the structure of photosynthetic proteins and enzymes. It is also essential for their defence system as an enzyme antioxidant cofactor^9^ and plays a key role in cell division^10^. Consumption of high manganese (Mn) concentrations can cause neurodegenerative disorders, cardiovascular toxicity, and liver damage^8^.

Zinc (Zn), one of the most mobile HMs, is present in the soils in free and complex ionic forms. Zn plays an important role in plant metabolism. It is also important in the activation of many enzymes included in the protein synthesis, cell membrane stabilization, auxin synthesis, and pollen formation^9^. Ingestion of high zinc levels results in neurotoxic^11^. Zinc is not generally considered to cause cancer^12^.

Copper (Cu) occurs naturally as a pure metal^13^. In plants, Cu is a component of several enzyme systems involved in oxidative stress responses, is involved in photosynthetic electron transport, mitochondrial respiration, and helps in the metabolism of lignin, carbohydrates, and proteins in plants^9^. Toxic effects of copper are associated with neurodegenerative diseases, diabetes^8^, acne, alopecia, autism, cystic fibrosis, hypothyroidism^14^.

Nickel (Ni) is essential for proper plant growth and development. However, at high levels, nickel alters plant metabolic activities, inhibits enzymatic activity, photosynthetic electron transport, and chlorophyll biosynthesis. Human exposure to nickel (Ni) mainly concerns oral ingestion through water and food that may be contaminated with nickel. Ni as an immunotoxic and carcinogenic substance can cause various health effects such as contact dermatitis, cardiovascular disease, asthma, pulmonary fibrosis, and respiratory cancer^15^.

Lead (Pb) is a highly toxic element, bioaccumulative, and degraded or easily metabolized in the environment^16^. The plants have only about 0.005 to 0.13% of lead in the soil solution. Nevertheless, food is a significant source of Pb exposure, and the potential risk to the population may be due to the bioaccumulation of Pb in the edible vegetable^4^. Lead exposure can cause plumbism, anaemia, nephropathy, gastrointestinal colic, and central nervous system symptoms^5^. Neurological symptoms include ataxia, encephalopathy, seizure, swelling of the optic nerve, disorder of consciousness^17^.

Cadmium (Cd) generally has a high soil bioavailability. It has higher mobility in plants compared to other heavy metals^18^, and in addition to reduced nutrient intake, causes chlorosis, necrosis, and growth retardation of roots and shoots^19^, can inhibit seed germination, and reduce the number of leaves per plant. It is toxic to plants even at low concentrations^20,21^. Cadmium is primarily toxic to the kidneys and can cause them to fail^22^. Another target organ for Cd is the liver^23^. In addition, it is involved in bone diseases, lung edema, liver damage, anaemia, and hypertension and is the cause of Itai-itai disease^18^. Cadmium is classified as a human carcinogen based on working studies (group 1)^24^.

Mercury (Hg) is recognized as a toxic, persistent, and mobile contaminant that does not degrade in the environment and is the only element in the periodic table with its own environmental convention, i.e., the Minamata Convention on Mercury^25^. Hg exposure can also reduce photosynthesis, transpiration rate, and water absorption, and chlorophyll synthesis^26^. Mercury is toxic to humans in all its primary forms, with the most toxic being methylmercury. Mercury is considered by WHO to be one of the top ten chemicals or groups of chemicals of major public health concern^27^.

The aim of this study was to (i) assess the impact of old environmental burdens on soil quality by soil indicators using soil risk contamination factors, (ii) determine the ability of Fe, Mn, Zn, Cu, Ni, Pb, Cd, and Hg to accumulate in crops (*Solanum tuberosum,* L., *Solanum esculentum,* L., and *Daucus carota*), and finally (iii) assess the risk of consuming crops grown in soils with increased content of these risk elements.

## Material and methods

### Study area

The Spiš region is one of the most burdened and hygienically defective areas due to mining activities that have been expanded in the past. The predominant contaminants are heavy metals^2^.

The area of interest is situated in the locality of the village Poráč, in the Slovak Ore Mountains (Volovské Hills–Hnilecké Hills). In terms of geological construction, the area is built by a diverse range of rocks. It is situated in the boundary of massifs formed by a complex of Mesolithic rocks (Gutenstein limestones) and the occurrence of sandstones, conglomerates, clayey shales, phyllites, and volcanics. Limestone complexes occur mainly in the eastern part of the territory, and other rocks make up a substantial majority of the area^28^. This diverse geological structure is followed by land cover. Modal rendzinas have been formed in the area of limestones, and cambisol-like rendzina has been formed in places where limestones pass into other rocks. The other area consists exclusively of modal acid cambisols^29^.

Rendzinas have a shallow humus horizon, but the humus content tends to be high, as calcium carbonate and humic substances form complexes. Below the humus horizon, a compact rock immediately occurs. The soil reaction of these soils is alkaline, the sorption capacity is high. Cambisol is characterized by a Cambic Bv horizon, which has a distinct brown color caused by Fe oxidation. The Cambic horizon is below the shallow humus horizon. Different types of rocks are the substrate for these soils, and therefore, the properties of these soils tend to be very diverse. Under forest stands, there is usually a higher amount of humus, but the values of the soil reaction range from weakly acidic to strongly acidic soil reaction. The sorption capacity is medium, the soil profile is skeletal and permeable to rainwater^30^.

### Sampling and sample processing

**Figure 1:**
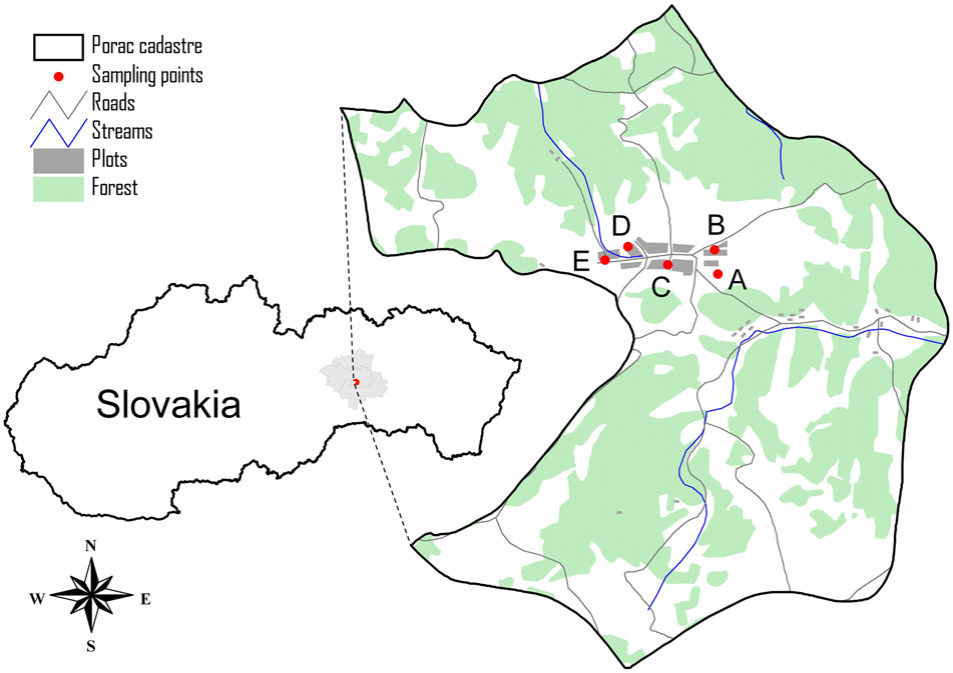
Map of the studied area and sampling sites.

Soil samples were collected in the cadastre of Poráč, which is a part of the Spiš region (Fig. 1). Map with sample sites was made with ArcView 3.2. Potato (*Solanum tuberosum,* L.), tomato (*Solanum esculentum,* L.), and carrot (*Daucus carota*) samples were also taken from a pre-determined five plots (A: 48.883396, 20.728739; B: 48.884139, 20.731591; C: 48.883378, 20.723065; D: 48.883471, 20.716939, E: 48.884733, 20.719931). Each soil and plant sample consisted of an average sample from 3 points (approx. 0.5 kg from one sampling point) from an area of approx. 400–700 m^2^. Soil samples were taken at a horizon of 0–0.1 m into pedological probe GeoSampler by Fisher.

Organic impurities (leaves, roots) and debris were removed from the soil samples before drying. After drying, the samples were ground (grinding machine VEB Thurm ZG 1) to fine earth (average particle size 0.125 mm), in which the contents of risk metals were determined. The soil samples were stored in polyethylene bags until analysis.

Samples of plant material were mechanically cleaned from organic and inorganic impurities immediately after collection. Subsequently, they were washed with distilled water, sliced, and dried to constant weight at 45 °C. After drying, the samples were homogenized (IKA A10 basic, 30 s., 25,000 rpm). All samples were stored in polyethylene bags until analysis.

### Chemical analysis

The contents of risk elements were determined in soil samples using Flame AAS method (Fe, Mn, Zn, Cu, Ni) and Graphite Furnace AAS method (Cd, Pb) (VARIAN AASpectr DUO 240FS/240Z/UltrAA equipped with a D2 lamp background correction system, using an air-acetylene flame, Varian, Ltd., Mulgrave, VIC, AUS). Total Hg content was determined using the Cold Vapour AAS method (AMA 254, Altec s.r.o, Prague, CZE). After microwave digestion (70 min, MARS X-Press 5, CEM Corp., Matthews, NC, USA), the total contents of risk elements, including all metal forms with exception of silicate forms in soil extract by *aqua regia* (1 g fine earth + 10 mL *aqua regia*, HNO_3_, HCl; Merck, Germany) were determined. In soil extract by NH_4_NO_3_ (c = 1 mol/L, NH_4_NO_3_; Merck, Germany), the contents of risk metals in their bioavailable form were determined.

Mineralization of plant samples was performed using a closed microwave digestion system (Mars X-Press 5) with conc. HNO_3_. The contents of risk elements in the plant material were determined by F-AAS method (Fe, Mn, Zn, Cu, Ni), GF-AAS method (Cd, Pb), and CV-AAS method (Hg). Fe (Mn, Zn, Cu, Ni, Cd, Pb, and Hg) were determined at wavelength 241.8 (279.5, 213.9, 324.8, 232.0, 228.8, 217.0, and 253,65) nm, Limits of detection LOD were 0.2978 (0.0253, 0.0868, 0.0880, 0.3582, 0.0617, 0.0891, and 0.0190^–6^) mg/L and Limits of quantification LOQ were 0.4703 (0.0302, 0.1736, 0.0917, 0.5652, 0.1192, 0.1405, and 0.0391) mg/L. Repeatability of determination during analysis—deviation max. 3%. Gas flow: air 13.5 L/min, acetylene 2.0 L/min. The measured results were compared with multielemental standard for F-AAS and GF-AAS, resp. singleelemental standard for CV-AAS (Merck, Germany).

The contents of risk elements determined in soil samples were compared with limit and critical values according to^31^.

The contents of risk elements determined in plant samples were evaluated according to the maximum allowed amounts given by the valid legislation^32–35^. There are no maximum levels for other risk elements.

Soil contamination was assessed using indicators of soil contamination by risk elements, the risk of heavy metals entering the food chain by a bioaccumulation factor, and the impact of risk elements on human health was assessed on the basis of human health risk indicators.

### Indicators of soil contamination by risk elements

1. **Contamination factor** (${\mathrm{C}}_{\mathrm{f}}^{\mathrm{i}},$ single element contamination factor, Eq. 1):
  1 $${\mathrm{C}}_{\mathrm{f}}^{\mathrm{i}}=\frac{{\mathrm{C}}^{\mathrm{i}}}{{\mathrm{B}}^{\mathrm{i}}}$$
  is given by the ratio of the concentration of the given risk element in the soil (${\mathrm{C}}^{\mathrm{i}}$) and its background concentration (level of geochemical background; ${\mathrm{B}}^{\mathrm{i}}$)^36^.
2. **Degree of contamination** (C_deg_, Eq. 2):
  is the sum of contamination factors for all examined risk elements and represents the integrated pollution degree of the environment^37^.
  2 $${\mathrm{C}}_{\mathrm{deg}}= \sum \frac{{\mathrm{C}}^{\mathrm{i}}}{{\mathrm{B}}^{\mathrm{i}}}= \sum {\mathrm{C}}_{\mathrm{f}}^{\mathrm{i}}$$
3. **Geo-accumulation index** (I_geo_, Eq. 3):
  expresses differences in environmental contamination between current and pre-industrial concentrations^38,39^. The constant 1.5 (Eq. 3) was introduced to minimize the effect of potential differences in background values that could be attributed to rocky differences in sediments^40^.
  3 $${\mathrm{I}}_{\mathrm{geo}}= {\mathrm{log}}_{2}\left(\frac{{\mathrm{C}}^{\mathrm{i}}}{\mathrm{1,5}\times {\mathrm{B}}^{\mathrm{i}}}\right)$$
4. **Pollution load index** (PLI, Eq. 4):
  4 $$\mathrm{PLI}={(\mathrm{C}}_{\mathrm{f}1}^{\mathrm{i}}\times {\mathrm{C}}_{\mathrm{f}2}^{\mathrm{i}}\times {\mathrm{C}}_{\mathrm{f}3}^{\mathrm{i}}\times \dots \times {\mathrm{C}}_{\mathrm{fn}}^{\mathrm{i}}{)}^{\frac{1}{\mathrm{n}}}$$
  is defined as the n-th root of the multiplications of the contamination factor (${\mathrm{C}}_{\mathrm{f}}^{\mathrm{i}}$) of metals, is it an integrated approach of pollution load index of the hazardous elements^41^.
5. **Bioaccumulation factor** (BAF_i_, Eq. 5):
  5 $${\mathrm{BAF}}_{i}= \frac{{\mathrm{C}}_{\mathrm{p}}^{\mathrm{i}}}{{\mathrm{C}}_{\mathrm{s}}^{\mathrm{i}}}$$
  is calculated from the ratio of the concentration of risk element in the plant (${\mathrm{C}}_{\mathrm{p}}^{\mathrm{i}}$) and in the soil (${\mathrm{C}}_{\mathrm{s}}^{\mathrm{i}}$)^3,5^.
6. **Estimated daily intake** (EDI, Eq. 6):
  is given by the concentration of the element in the food (C, mg/kg FW), the daily food intake (F_IR_, g/day), and the reference body weight (BWa, 70 kg)^42^.
  6 $$\mathrm{EDI}= \frac{\mathrm{C}\times {\mathrm{F}}_{\mathrm{IR}}}{\mathrm{BWa}}$$
7. **Chronic Daily Intake** (CDI, Eq. 7):
  was determined by a modified method according to Antoine et al.^42^, and Onyele and Anyanwu^43^. CDI is the daily dose of risk elements (mg/kg/day), C is the concentration of heavy metals in the food (mg/kg FW), E_FR_ is the exposure frequency to the trace element (daily/year), ED is the duration of exposure (70 years), AT is the average time (daily for 70 years), and 10^-3^ is the unit conversion factor
  7 $$\mathrm{CDI}= \frac{\mathrm{C}\times {\mathrm{F}}_{\mathrm{IR}}\times {\mathrm{E}}_{\mathrm{FR}}\times {\mathrm{E}}_{\mathrm{D}}}{\mathrm{BWa}\times \mathrm{AT}}\times {10}^{-3}$$
8. **Target hazard quotient** (THQ, Eq. 8):
  is given by the ratio of the daily dose of risk elements to which the consumer may be exposed, and the reference dose of risk elements that can be taken daily for a long period without health risk (RfD, mg/g/day)^5^.
  8 $$\mathrm{THQ}= \frac{\mathrm{CDI}}{\mathrm{RfD}}$$
9. **Hazard index** (HI, Eq. 9):
  9 $$\mathrm{HI}= \sum \mathrm{THQ}$$

Hazard index (HI) is the sum of the individual target hazard quotient (THQ) of elements evaluated for each type of food^42^.

### Statistical analyses

Results were evaluated using descriptive statistical analysis (Microsoft Excel, Redmond, WA, USA) and analysis of variance (One-Way ANOVA multi-range tests, method: 95.0 percent LSD) using Statgraphics statistical software (Centurion XVI.I, USA).

## Results and discussion

### Soil

#### Content of heavy metals in soil

The heavy metal contents in the soil were determined in the soil extract by *aqua regia* (Table 1). Mineralization with *aqua regia* is a method of determining the content of metals in the soil, which dissolves most of the soil constituents except those strongly bound in silicate minerals. In this way, all elements that are likely to become bioavailable in the long term are determined. Their content is sometimes referred to as "pseudototal" (determined in *aqua regia*)^44^.

**Table 1:** The content of pseudototal forms of heavy metals in the soil (mg/kg).

Plot	Fe	Mn	Zn	Cu	Ni	Pb	Cd	Hg
**A**								
Mean	31634^b^	2211^c^	151^b^	77.4^b^	39.5^ab^	49.5^a^	2.90^b^	6.60^a^
STDEV	1101	146	20.3	13.6	1.67	4.93	0.224	2.33
Min	30,876	2096	135	65.3	38.5	43.9	2.70	4.13
Max	32,897	2375	174	92.1	41.4	53.3	3.14	8.78
**B**								
Mean	34809^bc^	1124^ab^	185^c^	219^d^	63.0^c^	48.8^a^	3.34^c^	81.8^c^
STDEV	3019	81.3	4.28	26.5	3.06	2.42	0.360	6.31
Min	32,788	1052	182	201	59.8	46.0	2.98	75.3
Max	38,279	1212	190	249	65.9	50.5	3.70	87.9
**C**								
Mean	38653^c^	1900^c^	104^a^	164^c^	44.5^b^	44.9^a^	3.9^d^	37.8^b^
STDEV	4539	785	15.6	17.7	18.4	4.48	0.13	0.154
Min	33,602	1025	90.2	148	32.8	41.0	3.8	37.7
Max	42,390	2542	121	183	65.7	49.8	4.0	38.0
**D**								
Mean	20637^a^	541^a^	166^bc^	16.7^a^	25.5^a^	49.2^a^	2.45^a^	42.7^b^
STDEV	555	27.5	5.75	0.200	0.100	2.35	0.035	3.05
Min	20,082	513	160	16.5	25.4	46.8	2.41	39.7
Max	21,192	568	172	16.9	25.6	51.5	2.48	45.8
**E**								
Mean	38142^bc^	1726^bc^	362^d^	98.8^b^	44.4^b^	44.2^a^	4.29^e^	9.68^a^
STDEV	6047	315	31.8	30.3	5.00	5.15	0.045	2.04
Min	32,096	1410	331	68.5	39.4	39.0	4.24	7.64
Max	44,189	2041	394	129	49.4	49.3	4.33	11.7
Limit value*	550	–	150	60.0	50.0	70.0	0.700	0.500

*For soil extract by *aqua regia* according to legislation valid in the Slovak Republic^31^.

^a–d^Statistically significant differences between plots, P value < 0.0 of One-Way ANOVA analysis.

The heavy metal contents have been compared to their limit values (the maximum permissible contents of hazardous substances in agricultural land^31^). It is possible to state that the limit value was exceeded several times in the case of Fe (Cd and Hg). The contents of these elements, even at their lowest concentrations, were higher than limit values 36.5 (34.4 and 8.3, respectively). The highest concentrations of Fe (Zn, Cu, Ni, Cd, and Hg) exceeded limit values 80.3 (2.6, 4.2, 1.3, 61.9, and 175.8, respectively) times. The lead concentration in its pseudototal form was lower than its limit value in all forms in soil. There is no limit value for Mn according to Slovak legislation. There are statistically significant differences between the contents of heavy metals in the soils of individual plots. Based on the obtained results, plot B is the most contaminated (Cu, Ni, Hg).

Many studies pointed to the extraction and processing of heavy metals as one of the most important anthropogenic sources of soil contamination. Critical heavy metal pollution is caused by mining activities in the Guiyang area (China). The average contents of Zn, Pb, Cd, and Cu in the soil were 508.6, 384.8, 7.53, and 356 mg/kg, respectively^45^. Due to mining activities and inadequate disposal of waste materials in mining areas of Gifurwe, Burera district of Northern Rwanda (also known as the tungsten belt), high concentrations of heavy metals, especially As (531 mg/kg), Cr (130 mg/kg), and Pb (56 mg/kg), are present in agricultural soils^46^. High concentrations of Mn (1008–2007), Zn (63–140), Cu (76.3–252), Ni (44–84), Pb (44 mg/kg), and Sr (46–51) mg/kg were recorded in soil samples in the Falansa mining area and the Olode area (Pegmatite mining area) in Nigeria^47^. Due to the mining of lead and zinc ore in Kishnica (Kosovo), the concentrations of heavy metals in the soil samples exceed the standard values. The average content of Fe (6009.81), Pb (3106.49), Ni (277.07), and Cd (3.49) mg/kg was significantly (p < 0.01) higher than in the soil from the uncontaminated area of Koliq^48^.

Mobile (bioavailable) forms of heavy metals are crucial for assessing soil hygiene (biotoxicity). Their concentrations are given in Table 2.

**Table 2:** The content of bioavailable forms of heavy metals in soil (mg/kg).

plot	Fe	Mn	Zn	Cu	Ni	Pb	Cd
**A**							
Mean	0.493^a^	7.603^a^	0.213^a^	0.397^b^	0.237^a^	0.320^a^	0.136^bc^
STDEV	0.060	3.784	0.040	0.071	0.021	0.010	0.008
Min	0.430	4.240	0.190	0.320	0.220	0.310	0.127
Max	0.550	11.700	0.260	0.460	0.260	0.330	0.142
**B**							
Mean	0.910^b^	6.27^a^	0.280^a^	0.940^d^	0.260^ab^	0.410^ab^	0.153^c^
STDEV	0.046	0.314	0.014	0.047	0.013	0.021	0.008
Min	0.865	5.96	0.266	0.893	0.247	0.390	0.145
Max	0.956	6.58	0.294	0.987	0.273	0.431	0.161
**C**							
Mean	0.407^a^	14.6^a^	0.440^a^	0.660^c^	0.230^a^	0.313^a^	0.122^ab^
STDEV	0.035	10.6	0.374	0.131	0.030	0.105	0.025
Min	0.370	2.98	0.190	0.540	0.200	0.210	0.098
Max	0.440	23.8	0.870	0.800	0.260	0.420	0.148
**D**							
Mean	2.33^c^	24.7^b^	10.8^b^	0.090^a^	0.340^c^	0.325^a^	0.108^a^
STDEV	0.400	1.24	0.390	0.010	0.040	0.045	0.013
Min	1.93	23.4	10.5	0.080	0.300	0.280	0.094
Max	2.73	25.9	11.2	0.100	0.380	0.370	0.121
**E**							
Mean	0.795^ab^	10.10^a^	0.450^a^	0.535^c^	0.295^bc^	0.430^b^	0.161^c^
STDEV	0.275	1.77	0.008	0.055	0.015	0.030	0.005
Min	0.520	8.33	0.442	0.480	0.280	0.400	0.156
Max	1.07	11.87	0.457	0.590	0.310	0.460	0.166
Critical value*	–	–	2.0	1.0	1.5	0.1	0.1

*For soil extract by NH_4_NO_3_ according to legislation valid in the Slovak Republic^31^.

^a–d^Statistically significant differences between plots, P value < 0.05 of One-Way ANOVA analysis.

Critical values for bioavailable forms of heavy metals were exceeded in the case of Pb. Its lowest content was 2.1 times higher than the specified critical value (0.1 mg/kg). The highest contents of Zn (Cd) exceeded the set critical values by 5.6 (1.7) many times. No critical values are set for Fe and Mn.

There are statistically significant differences between the contents of heavy metals in the soils of individual plots (Tables 1, 2).

#### Indicators of soil contamination by risk elements

The pollution indices can be divided into six groups for different calculation purposes and can provide information on: (i) the individual pollution levels from each of the heavy metals analyzed (I_geo_, PI, C_f_); (ii) the scale of the total pollution (PI_sum_, PI_Nemerow_, PLI, PI_ave_, mCd, PI_Vector_, C_deg_, PIN and SCI); (iii) the heavy metal sources (EF and MEC); (iv) the potential environmental risk (RI and MERMQ); (v) areas with the highest potential risk of heavy metal accumulation (ExF); and (vi) the ability of the horizon surface to accumulate heavy metals (BGI)^49^.

We evaluated the degree of soil contamination by analysis of Contamination factor, Degree of contamination, Pollution load index, and Geo-accumulation index.

##### Contamination factor (C_f_), Degree of contamination (C_deg_):

Background values according to Linkeš et al.^50^ were used to calculate ${\mathrm{C}}_{\mathrm{f}}^{\mathrm{i}}$. The value of ${\mathrm{B}}^{\mathrm{i}}$ for manganese and iron is not stated in this publication, therefore the value according to He et al.^51^ and Demková et al.^52^, respectively was used (Table 3).

**Table 3:** The background values of risk elements in mg/kg.

	Fe	Mn	Zn	Cu	Ni	Pb	Cd	Hg
B_i_	530^3^	400^2^	64.26^1^	22.595^1^	12.79^1^	24.87^1^	0.285^1^	0.075^1^

^1^According to Linkeš et al.^50^.

^2^According to He et al.^51^.

^3^According to Demková et al.^52^.

The values of C_f_ and C_deg_ for individual heavy metals are given in Table 4.

**Table 4:** Contamination factor (C_f_) and degree of contamination (C_deg_).

plot	C_f_								C_deg_
	Fe	Mn	Zn	Cu	Ni	Pb	Cd	Hg	
**A**									
Mean	59.7	5.53	2.35	3.43	3.09	1.99	10.2	88.0	174
STDEV	2.08	0.365	0.316	0.601	0.131	0.198	0.785	31.1	31.9
Min	58.3	5.24	2.09	2.89	3.01	1.77	9.47	55.1	141
Max	62.1	5.94	2.70	3.31	3.24	2.06	10.0	117	205
**B**									
Mean	65.7	2.81	2.88	9.67	4.92	1.96	11.7	1091	1191
STDEV	5.70	0.203	0.067	1.17	0.239	0.097	1.26	84.2	86
Min	61.9	2.63	2.82	8.88	4.68	1.85	10.5	1004	1098
Max	72.2	3.03	2.96	11.0	5.15	2.00	13.0	1172	1269
**C**									
Mean	72.9	4.75	1.61	7.26	3.48	1.81	13.7	504	609
STDEV	8.56	1.96	0.243	0.784	1.44	0.180	0.464	2.05	8.37
Min	63.4	2.56	1.40	6.54	2.56	1.65	13.2	502	601
Max	80.0	6.35	1.88	8.09	5.14	2.00	14.1	506	617
**D**									
Mean	38.9	1.35	2.58	0.739	1.99	1.98	8.58	570	626
STDEV	1.05	0.069	0.089	0.009	0.008	0.094	0.123	40.7	41.9
Min	37.9	1.28	2.49	0.730	1.99	1.88	8.46	529	584
Max	40.0	1.42	2.67	0.748	2.00	2.07	8.70	611	668
**E**									
Mean	72.0	4.31	5.64	4.37	3.47	1.78	15.0	129	236
STDEV	11.4	0.789	0.495	1.34	0.391	0.207	0.158	27.2	14.4
Min	60.6	3.53	5.14	3.03	3.08	1.57	14.9	102	221
Max	83.4	5.10	6.13	5.71	3.86	1.98	15.2	156	250

The average C_f_ values for Fe, Cd, and Hg ranged from 8.58 (Cd, plot D) to 1091 (Hg, plot B). Based on these data, very high contamination of each of the mentioned elements can be stated for all plots. Plot C and B also showed a very high contamination degree of Cu, soils of plots A, D, E are slightly (1 ≤ ${\mathrm{C}}_{\mathrm{f}}^{\mathrm{i}}$<3) to substantially (3 ≤ ${\mathrm{C}}_{\mathrm{f}}^{\mathrm{i}}$<6) contaminated. Also, in the case of Mn, Zn, and Ni, C_f_ is in the range of 1 – 6. In the case of Pb, the C_f_ value was from 1.78 to 1.99, which indicates slight contamination with this element. The classification, according to Hakanson^53^, was used to assess the degree of contamination by individual heavy metals (Table 5), which was also used by other authors in their work^36,54,55^. In China, the C_f_ has been accepted as a pollution index (PI), which is often assessed by comparing metal concentrations with relevant environmental directives or concerning relevant background values. The contamination level is classified as follows: uncontaminated (PI < 1), Moderately contaminated (1 ≤ PI < 3), Considerable contamination (3 ≤ PI < 6), high contaminated (6 ≤ PI < 12), very high contaminated (12 < PI)^56^.

**Table 5:** Contamination factor (${\mathrm{C}}_{\mathrm{f}}^{\mathrm{i}}$)^53^, Degree of contamination (C_deg_)^57^.

${\mathrm{C}}_{\mathrm{f}}^{\mathrm{i}}$	Contamination degree of individual metal	C_deg_	Contamination degree of the environment
${\mathrm{C}}_{\mathrm{f}}^{\mathrm{i}}$<1	Low	C_deg_ < 5	Low contamination
1 ≤ ${\mathrm{C}}_{\mathrm{f}}^{\mathrm{i}}$<3	Moderate	5 ≤ C_deg_ < 10	Moderate contamination
3 ≤ ${\mathrm{C}}_{\mathrm{f}}^{\mathrm{i}}$<6	Considerable	10 ≤ C_deg_ < 20	Considerable contamination
${\mathrm{C}}_{\mathrm{f}}^{\mathrm{i}}$≥6	Very high	C_deg_ ≥ 20	Very high contamination

Based on the assessment of soil contamination using the degree of contamination C_deg_, the soils of all plots can be classified as very high contaminated (Table 4). This degree of contamination would also be if soils were assessed only on the basis of the presence of Fe and Hg, in the case of Cd, it would be considerable contamination. That means that these three elements contributed most significantly to soil contamination on individual plots. On the other hand, if only the presence of Mn, Zn, Cu, Ni, and Pb were evaluated, plot B could also be classified as very high contaminated (C_deg_ = 22.2). A scale, according to Luo et al.^57^ was used to evaluate the degree of contamination (C_deg_) (Table 5). Even if a wider range scale was used in the evaluation (C_deg_ ≥ 32: very high contamination^53^, resp. C_deg_ > 32: high contamination^1^, the soils of all plots would be classified as very high contaminated. Even if the soils were evaluated only on the basis on the presence of Fe and Hg.

##### Geo-accumulation index (I_geo_), pollution load index (PLI)

Geo-accumulation index is used as a quantitative index for the degree of heavy metal contamination in the deposit or other materials^58^. Pollution load index provides information on the total toxicity level of trace elements in a given sample and indicates how many times trace element concentrations in soil exceed background concentrations. This parameter can be used to determine the level of environmental pollution^40^. PLI was calculated as the eight root of the product (multiple) of the C_f_ concentration of the eight analyzed heavy metals. I_geo_ and PLI values are given in Table 6.

**Table 6:** Geo-accumulation index (I_geo_), Pollution load index (PLI).

plot	I_geo_								PLI
	Fe	Mn	Zn	Cu	Ni	Pb	Cd	Hg	
**A**									
Mean	5.31	1.88	0.64	1.18	1.04	0.402	2.76	5.81	1.67
STDEV	0.050	0.094	0.190	0.250	0.060	0.148	0.110	0.555	0.007
Min	5.28	1.80	0.48	0.95	1.00	0.232	2.66	5.20	1.66
Max	5.37	1.99	0.85	1.44	1.11	0.512	2.88	6.29	1.68
**B**									
Mean	5.45	0.90	0.94	2.68	1.71	0.385	2.96	9.50	1.69
STDEV	0.122	0.104	0.033	0.170	0.070	0.073	0.156	0.112	0.018
Min	5.37	0.81	0.91	2.57	1.64	0.302	2.80	9.39	1.67
Max	5.59	1.01	0.98	2.88	1.78	0.437	3.11	9.61	1.71
**C**									
Mean	5.60	1.56	0.09	2.27	1.14	0.263	3.19	8.39	1.71
STDEV	0.174	0.695	0.214	0.155	0.554	0.142	0.049	0.006	0.026
Min	5.40	0.77	− 0.10	2.12	0.77	0.136	3.14	8.39	1.68
Max	5.74	2.08	0.33	2.43	1.78	0.42	3.23	8.40	1.73
**D**									
Mean	4.70	− 0.15	0.78	− 1.02	0.41	0.397	2.52	8.57	1.58
STDEV	0.039	0.073	0.050	0.017	0.006	0.069	0.021	0.103	0.005
Min	4.66	− 0.23	0.73	− 1.04	0.40	0.327	2.50	8.46	1.58
Max	4.74	− 0.08	0.83	− 1.00	0.42	0.465	2.54	8.67	1.59
**E**									
Mean	5.57	1.51	1.91	1.50	1.20	0.236	3.33	6.41	1.71
STDEV	0.231	0.267	0.127	0.458	0.163	0.169	0.015	0.310	0.034
Min	5.34	1.23	1.78	1.02	1.04	0.064	3.31	6.08	1.67
Max	5.80	1.77	2.03	1.93	1.36	0.402	3.34	6.70	1.74

A seven-level scale of contamination was used to assess soil contamination based on I_geo_^58^ (Table 7). Seven I_geo_ classes, but with different expressions of the level of contamination are given by other authors^38,40,56^. According to the classification by Yakun et al.^58^, individual plots can be classified as not contaminated with Mn and Cu (plot D), lightly contaminated with Pb and Zn (all plots except plot E with Zn, which was slightly moderate contaminated), Mn (plot B), and Ni (plot D), slightly moderate contaminated with Mn (plots A, C, E), Cu (plots A, E), and Ni (all plots except D), moderate contaminated with Cu (plots B, C), and Cd (plots A, B, D), slightly heavy contaminated with Cd (plots C, E), and extremely heavy contaminated with Fe and Hg (except plot D, which was heavily contaminated with Fe). Even though 57.5% of I_geo_ were ≤ 2, these soils pose a risk mainly due to mercury. The soils around the mining areas are seriously contaminated with heavy metals emitted from mining activities. Similar results were reported by Li et al.^38^.

**Table 7:** Pollution load index^40^, Geo-accumulation index (I_geo_)^58^.

Contam. degree	I_geo_ value	Contamination level	PLI class	PLI value	Pollution level
0	I_geo_ < 0	No contamination			
1	0 < I_geo_ ≤ 1	Light contamination	1	0 < PLI ≤ 1	Unpolluted
2	1 < I_geo_ ≤ 2	Slightly moderate contamination	2	1 < PLI ≤ 2	Moderately polluted to unpolluted
3	2 < I_geo_ ≤ 3	Moderate contamination	3	2 < PLI ≤ 3	Moderately polluted
4	3 < I_geo_ ≤ 4	Slightly heavy contamination	4	3 < PLI ≤ 4	Moderately to highly polluted
5	4 < I_geo_ ≤ 5	Heavy contamination	5	4 < PLI ≤ 5	Highly polluted
6	I_geo_ > 5	Extremely heavy contamination	6	5 ≤ PLI	Very highly polluted

*Contam. degree* contamination degree.

The application of PLI was used to evaluate environmental risk caused by contaminated soil. Pollution load index ranged from 1.58 (plot D) to 1.71 (plots C, E). The results showed that all five plots were from moderately polluted to unpolluted. This assessment is based on a six-level classification of pollution levels, according to Abowaly et al.^40^ (Table 7). Qarri et al.^1^ added PLI class 0—background level to the classification. On the other hand, Varol^87^ mentioned a simple assessment of the level of heavy metal pollution, resp. deterioration of soil conditions due to the accumulation of heavy metals. If PLI < 1, no metal contamination occurs, and PLI > 1 indicates deterioration of soil quality. According to this assessment, all plots were deteriorated in terms of heavy metal contamination.

### Plant

#### The content of heavy metals in soil

The contents of Fe, Zn, Mn, Cu, Ni, Cd, Pb, and Hg were determined in potatoes, carrots, and tomatoes. Samples of the given crops were taken from all investigated plots, except for tomatoes, which were not grown on plots D and E. Heavy metal concentrations are expressed in mg/kg dry weight (DW), for comparison with maximum levels for contaminants in foodstuff (according to Slovak/European regulations) the concentration values are given converted to mg/kg fresh weight (FW) (Table 8). No maximum levels are set for Fe, Mn, and Zn.

**Table 8:** The content of heavy metals in plant materials (mg/kg DW).

Crop/plot	Fe	Mn	Zn	Cu	Ni	Pb	Cd	Hg
**Potato/A**								
Mean	74.9^a^	6.50^a^	12.5^a^	14.3^a^	BDL	2.76^b^	BDL	0.150^c^
STDEV	5.24	0.455	0.874	0.998		0.194		0.0105
FW	17.74	1.54	2.96	3.38		0.655		0.0356
**Potato/B**								
Mean	254^c^	16.1^c^	19.3^c^	13.1^a^	0.552^d^	1.07^a^	BDL	0.190^d^
STDEV	17.8	1.13	1.35	0.914	0.0387	0.075		0.0133
FW	60.2	3.83	4.56	3.09	0.131	0.253		0.0450
**Potato/C**								
Mean	285^d^	19.9^d^	15.5^b^	17.0^b^	0.259^c^	2.58^b^	BDL	0.444^e^
STDEV	19.9	1.40	1.1	1.19	0.0181	0.181		0.0311
FW	67.5	4.72	3.67	4.03	0.0613	0.611		0.105
**Potato/D**								
Mean	72.9^a^	7.94^ab^	20.9^c^	18.2^b^	0.001^a^	2.84b	BDL	0.108^b^
STDEV	5.11	0.556	1.46	1.28	0.0001	0.198		0.0076
FW	17.3	1.88	4.95	4.32	0.00034	0.672		0.0257
**Potato/E**								
Mean	99.5^b^	8.98^b^	25.2^d^	25.1^c^	0.100^b^	3.48^c^	BDL	0.0630^a^
STDEV	6.96	0.629	1.76	1.75	0.0070	0.244		0.0044
FW	23.6	2.13	5.97	5.94	0.0238	0.825		0.0149
ML	–	–	–	3.00^4^	0.5^4^	0.1^2^	0.1^1^	0.02^3^
**Carrot/A**								
Mean	87.9^b^	14.4^c^	22.4^ab^	21.5^b^	1.14^a^	2.97	0.0139	0.110^a^
STDEV	6.15	1.01	1.57	1.51	0.079	0.208	0.0010	0.0077
FW	10.5	1.73	2.69	2.58	0.136	0.356	0.0017	0.0132
**Carrot/B**								
Mean	35.3^a^	6.25^a^	20.7^a^	11.1^a^	5.21^d^	1.70	BDL	0.180^a^
STDEV	2.47	0.44	1.45	0.78	0.365	0.119		0.0126
FW	4.23	0.750	2.48	1.34	0.625	0.205		0.0216
**Carrot/C**								
Mean	80.2^ab^	11.1^bc^	28.7^c^	23.4^bc^	3.63^c^	2.29	BDL	0.108^a^
STDEV	5.61	0.78	2.01	1.64	0.254	0.160		0.0076
FW	9.62	1.34	3.44	2.81	0.436	0.274		0.0130
**Carrot/D**								
Mean	103^b^	7.55^ab^	25.0^bc^	24.5^c^	0.942^a^	2.48	BDL	0.147^a^
STDEV	7.24	0.53	1.75	1.71	0.066	0.174		0.0103
FW	12.4	0.906	3.00	2.94	0.113	0.298		0.0177
**Carrot/E**								
Mean	894^c^	59.8^d^	51.3^d^	23.7^bc^	2.42^b^	ND	0.0589	1.23^b^
STDEV	62.6	4.19	3.59	1.66	0.169		0.0041	0.0864
FW	107	7.18	6.15	2.85	0.290		0.0071	0.148
ML	–	–	–	10.0^4^	2.5^4^	0.1^2^	0.1^1^	0.03^3^
**Tomato/A**								
Mean	85.9^a^	14.5^a^	20.1^ab^	17.4^a^	1.20^b^	2.48^c^	BDL	0.055^a^
STDEV	6.01	1.01	1.41	1.22	0.084	0.173		0.0038
FW	5.41	0.912	1.27	1.10	0.0759	0.156		0.0034
**Tomato/B**								
Mean	234^c^	20.9^b^	22.4^b^	28.8^b^	2.44^c^	0.286^a^	BDL	0.155^c^
STDEV	16.4	1.47	1.57	2.01	0.171	0.020		0.0109
FW	14.7	1.32	1.41	1.81	0.154	0.0180		0.0098
**Tomato/C**								
Mean	111^b^	16.3^a^	19.4^a^	20.4^a^	0.469^a^	1.28^b^	BDL	0.126^b^
STDEV	7.76	1.14	1.36	1.43	0.033	0.089		0.0088
FW	6.99	1.03	1.23	1.29	0.0295	0.0803		0.0079
ML	–	–	–	10.0^4^	2.5^4^	0.05^2^	0.02^1^	0.03^3^

*DW* dry weight, *FW* fresh weight (mg/kg), *ML* maximum levels (mg/kg FW), *BDL* below detection limit.

^1^According to EU^32^.

^2^According to EU^33^.

^3^According to Regulation SR^34^.

^4^According to Regulation SR^35^.

^a–e^Statistically significant differences between plots, P value < 0.0 of One-Way ANOVA analysis.

The maximum levels (ML) set for Cu, Ni, Pb Cd, and Hg have been exceeded for Cu, Pb, and Hg. The copper content was higher in all potato samples than ML (3 mg/kg FW). The lowest Cu content was in potatoes from plot B, the highest in potatoes from plot E, where the ML was exceeded by 97%. The lead content exceeded the limit value in potatoes and carrots (ML = 0.10 mg/kg FW) from all plots except carrots from plot E and tomatoes (ML = 0.05 mg/kg FW) from plots A and C. The highest exceedance of the ML was in the case of potatoes, while lead content in potatoes from plot E exceeded the ML up to 8.25 times. Lead has the second-lowest mobility in the soil horizon after mercury^44^. Nevertheless, the effect of a high concentration of its mobile form in the soil was demonstrated (Table 2). The mercury content was lower than ML (0.03 mg/kg FW) in tomatoes and carrots from all plots. In only one case (carrots from plot E), the ML was exceeded—by almost 400%. On the other hand, the lowest Hg concentration was determined in potatoes from this plot. In the samples from plot D (A, B, C), the concentration of Hg was 1.25 (1.78, 2.25, 5.25, respectively) times higher than the determined ML of mercury for potatoes (0.02 mg/kg FW).

Similarly, increased Pb, Zn, Hg, Cu, and Cd levels in carrots and potatoes were reported by Miller et al.^59^. Water and agricultural land contamination are caused by mining activity in Cerro Rico de Potosí (Southern Bolivia). Higher contents of Ni (0.1–2.5 mg/kg FW) and Hg (0.001–0.116 mg/kg FW) were determined by Yeganeh et al.^88^ in potatoes grown in Hamedan province (northwest of Iran). The Ni (Hg) content in soil was 26–140 (0.054–0.316) mg/kg. In vegetable samples grown locally in the suburban of Isfahan city (Iran), the content of Pb in carrots was up to 7.14 (Cd up to 2.91) mg/kg DW and in potatoes—Pb up to 7.14 (Cd up to 0.67) mg/kg DW^60^. Rayhan Khan et al.^61^, based on selected publications from 2015 to 2020, assessed heavy metal levels in more than 20 types of vegetables available in Bangladesh. The Mn (Fe, Ni, Cu, Zn, Cd, Pb) content was up to 210.70 (2362.56, 37.52, 45.00, 174.60, 240.00, 31.1, respectively) mg/kg. Compared to the FAO/WHO standard, the safe limit was exceeded for all listed elements except Mn.

#### Bioaccumulation factor (BAF)

Bioaccumulation factor refers to the ratio of heavy metal content in the plant to its content in the soil^62^, which reflects the plant’s ability to absorb heavy metal^58^. The BAF values, minimum and maximum values calculated for potatoes, carrots, and tomatoes grown on individual plots, are given in Table 9.

**Table 9:** Bioaccumulation factor (BAF).

Crop	Fe	Mn	Zn	Cu	Ni	Pb	Cd	Hg
**Potato**								
Min	2.251E−03	3.007E−03	0.0639	0.0651	NC	0.0214	NC	2.163E−03
Max	8.476E−03	0.0194	0.1284	1.0796	9.235E−03	0.0706	NC	0.0171
**Carrot**								
Min	1.076E−03	4.382E−03	0.1121	0.0541	0.0295	0.0000	NC	2.389E−03
Max	0.0278	0.0293	0.2869	1.4839	0.1037	0.0676	0.0136	0.1053
**Tomato**								
Min	2.775E−03	6.907E−03	0.1179	0.1155	0.0143	6.214E−03	NC	1.889E−03
Max	6.113E−03	0.0173	0.2156	0.1891	0.0370	0.0465	NC	0.0132

*NC* not calculated.

For plant and animal health, BAF > 1 is considered to be a hazardous value for heavy metals^63^. In the evaluation, we considered maximum BAF values for individual metals and crops. This value has been exceeded in the case of Cu in carrots and potatoes from plot D, which may already pose a risk to the consumer. In carrots, the highest BAF value was for Zn. Overall, these two metals (Cu and Zn) had the highest BAF values and, for other elements, there was no clear tendency to decrease BAF, but the lowest value was for Fe (carrot > potato > tomato). In this order, the BAF value decreased for Mn and Cu. Potatoes had the highest ability to accumulate Pb and tomatoes had the lowest. Authors^5,36,64^ confirmed different absorption capacities for different crops. However, Hu et al.^5^ further stated that Cd was most easily absorbed by crops, while Pb was identified as having the lowest accumulation in crops. In our case, the cadmium contents in all potato and tomato samples (and three carrot samples) were below the detection limit. Therefore, the BAF value for Cd was not evaluated.

### Human health risk assessment

#### Estimated daily intake (EDI)

The daily intake of metals depends on the concentration of metals in the food and the daily food consumption. In addition, a person's body weight can affect contaminant tolerance^65^. The estimated daily intake (EDI) of the heavy metals of interest (Fe, Mn, Zn, Cu, Ni, Pb, Cd, and Hg) were calculated based on their average concentration in the three vegetables (potato, carrot, tomato) and daily intake of the vegetable (Table 11). Data on the consumption of individual vegetables were obtained from Meravá^66^ (Table 10).

**Table 10:** Consumption of individual vegetables in Slovakia^66^.

	Carrot	Tomato	Potato
kg/year	12.9	18.5	53.6
g/day	35.34	50.68	146.85

The data obtained on the estimated daily intake showed that, in terms of heavy metal intake, potatoes are the riskiest of the monitored crops. The highest EDI values were in the case of Fe and decreased in the order of Fe > Zn = Cu > Mn > Pb > Hg. In the case of Ni and Cd, the highest EDI is through a carrot. EDI values were compared with TDI (TWI, PTWI, PMTDI, UL), which were expressed as tolerable daily intake from the baseline data for easier comparison (Table 11). For zinc, the value determined for pregnant and lactating women was used, and for lead, the PTWI value of 25 μg/kg bw/week was used for comparison. This data was used even though the CONTAM Panel concluded that the current PTWI of 25 μg/kg bw is no longer appropriate as there is no evidence for a threshold for critical lead-induced effects. In adults, children, and infants, the margins of exposures were such that the possibility of an effect from lead in some consumers, particularly in children from 1 to 7 years of age, cannot be excluded. Protection of children against the potential risk of neurodevelopmental effects would be protective for all other adverse effects of lead in all populations^67^. Furthermore, the CONTAM Panel set the TWI for inorganic mercury at 4 µg/kg body weight (bw), which is in accordance with EFSA^68^.

**Table 11:** Estimated daily intake (EDI, μg/day kg bw) and Tolerable daily intake (TDI, μg/day kg bw).

Crop	Fe	Mn	Zn	Cu	Ni	Pb	Cd	Hg
**Potato**								
Min	36.3	3.23	6.21	6.49	NC	0.531	NC	0.031
Max	142	9.91	12.5	12.5	0.275	1.73	NC	0.221
**Carrot**								
Min	2.14	0.379	1.25	0.675	0.057	0.000	NC	0.007
Max	54.2	3.63	3.11	1.48	0.316	0.180	0.0036	0.075
**Tomato**								
Min	3.92	0.660	0.887	0.795	0.021	0.013	NC	0.002
Max	10.7	0.955	1.02	1.31	0.111	0.113	NC	0,007
HGBV	PMTDI	TDI	UL	PTWI	TDI	PTWI	TWI	TWI
	0.8 mg/kg bw/day	0.06 mg/kg bw/day	25 mg/ person/day	3.5 mg/kg bw/week	2.8 μg/kg bw/day	25 μg/kg bw/week	2.5 μg/kg bw/week	4 µg/kg bw/week
TDI	800^1^	60^1^	357^1^	500^5^	2.8^4^	3.57^3^	0.357^1^	0.571^2^

*Bw* body weight, *NC* not calculated, *HGBV* health-based guidance values, *PMTDI* provisional maximum TDI, *UL* tolerable upper intake level, *TWI* tolerable weekly intake, *TDI* tolerable daily intake, *PTWI* provisional tolerable weekly intake, *HGBV(Zn)* recalculated from pregnant and lactating women.

^1^According to EFSA^83^.

^2^According to EFSA^68^.

^3^According to EFSA^67^.

^4^According to EFSA^75^.

^5^According to Mohamed et al.^84^.

Although the heavy metal contents were increased in all three crops and the limit values for these elements were exceeded for Cu, Pb, and Hg, the EDI values for all heavy metals were several times lower (min. TDI/EDI(Pb) = 2.06, max TDI/EDI(Cu) = 40), as determined by the tolerable daily intake. In thirteen Jamaican-grown food crops in which Al, As, Cd, and Pb were determined, the EDI for Pb (Cd) had lower values compared to our results: 0.004 (0.032), 0.002 (0.137), and 0.009 (0.116) μg/day/kg body weight^42^. Higher EDI values for cadmium (0.61–1.13) and, on the contrary, lower for Pb (0.27–0.47) calculated based on consumption of rice seeds of paddy fields in southwest of Iran reported Chamannejadian et al.^65^.

#### Chronic daily intake (CDI)

Chronic daily intake is the exposure expressed as the weight of the contacted substance per unit body weight per unit time, averaged over a long period (seven years to a lifetime)^69^. In our calculation, the period was expressed as 70 years. CDI values (Table 12) ranged from 2.495E−06 (Hg) to 0.1416 (Fe). The highest CDI values for all heavy metals except Ni and Cd were calculated for potatoes and corresponded to the highest EDI values (Table 11).

**Table 12:** Chronic daily intake (CDI, mg/kg/day).

Crop	Fe	Mn	Zn	Cu	Ni	Pb	Cd	Hg
**Potato**								
Min	0.0363	0.0032	0.0062	0.0065	NC	5.309E−04	NC	3.131E−05
Max	0.1416	0.0099	0.0125	0.0125	2.746E−04	1.731E−03	NC	2.209E−04
**Carrot**								
Min	0.0021	0.0004	0.0013	0.0007	5.709E−05	NC	NC	6.567E−06
Max	0.0542	0.0036	0.0031	0.0015	3.155E−04	1.798E−04	3.567E−06	7.482E−05
**Tomato**								
Min	0.0039	0.0007	0.0009	0.0008	2.139E−05	1.304E−05	NC	2.495E−06
Max	0.0107	0.0010	0.0010	0.0013	1.113E−04	1.130E−04	NC	7.092E−06

*NC* not calculated.

Based on estimates of current iron intake in European countries, the risk of side effects of high Fe intake from food sources, including fortified foods in some countries (but without supplements), is considered low for the general population. Foods rich in total iron include liver and offal, game, and beef; medium to high amounts of Fe also contain cereals, cereal products, and legumes. For the general population, food is the most important source of manganese exposure, but its concentrations vary considerably^70^. Cereals make up the major proportion of Mn in the diet (57%), followed by fruit, vegetables, nuts, and tea^71,72^. The main food groups contributing to zinc intake are meat and meat products, cereals and cereal-based products, milk, and dairy products^70,73^. Foods that contribute most to copper intake include cereals and cereal-based products, meat and meat products^74^, seafood, nuts, and seeds^70^. The main sources of Ni in the diet are cereals and cereal-based products, soft drinks (except milk-based beverages), sugar and confectionery, legumes, nuts and oilseeds, vegetables, and vegetable products. The CONTAM Panel concluded that dietary exposure probably represents the most important contribution to the overall Ni exposure in the general population^75^. The food groups that contributed to most of the dietary exposure to cadmium, mainly due to high consumption, were cereals and cereal-based products, vegetables, nuts, legumes, potatoes, meat, and meat products (EFSA 2009). Cereals and cereals, potatoes, vegetables, and tap water contributed to lead food exposure in the general European population but found no necessary data on bioaccumulation in the food chain^67,76^.

A survey of raw food in Germany in 1986 found that cereals, potatoes, vegetables, and fruits had average mercury concentrations of 0.005–0.05 mg/kg (ppm fresh weight), cocoa beans, tea leaves, and coffee beans contained average mercury concentrations of 0.005, 0.025, and 0.04 mg/kg, respectively^77^.

#### Target hazard quotient (THQ), hazard index (HI)

THQ is defined as the ratio of exposure to a toxic element and reference dose, which is the highest level at which no adverse health effects are expected. Reference dose (RfD) is specific for each trace element examined. The RfD values used in the calculation of THQ are given in Table 13. Hazard index (HI) is the sum of the individual target hazard quotients of the elements evaluated for each type of food^42^.

**Table 13:** Oral reference dose for risk elements.

Element	Fe	Mn	Zn	Cu	Ni	Pb	Cd	Hg
RfD (mg/kg/day)	0.024^2^	0.3^1^	0.037^1^	0.01^1^	0.02^1^	0.36^1^	0.0005^1^	0.0003^1^

^1^According to FRAMEWORK^85^.

^2^According to EPA^86^.

THQ values were calculated for each element and each vegetable as well as, HI values were calculated for all three vegetables.

**Figure 2:**
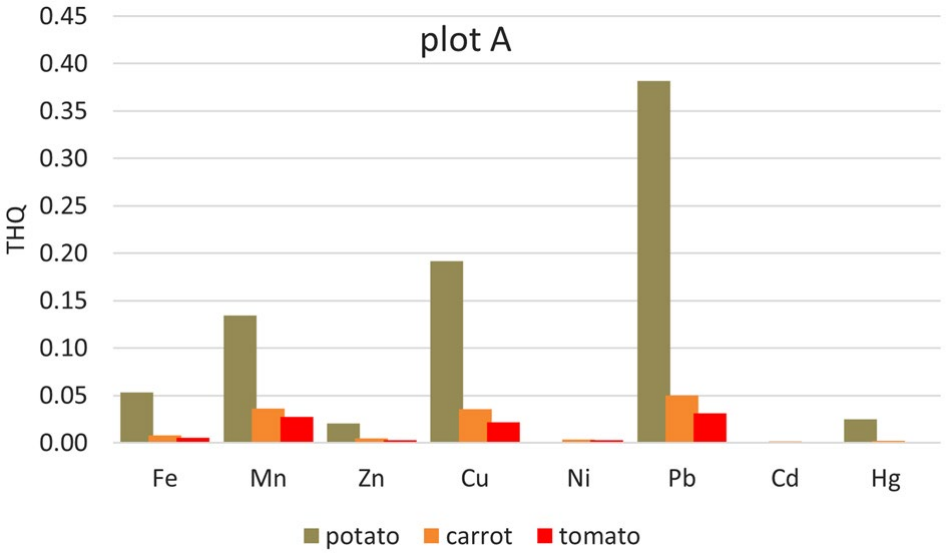
Target hazard quotient (THQ) of heavy metals in potatoes, carrots, and tomatoes grown in plot A.

**Figure 3:**
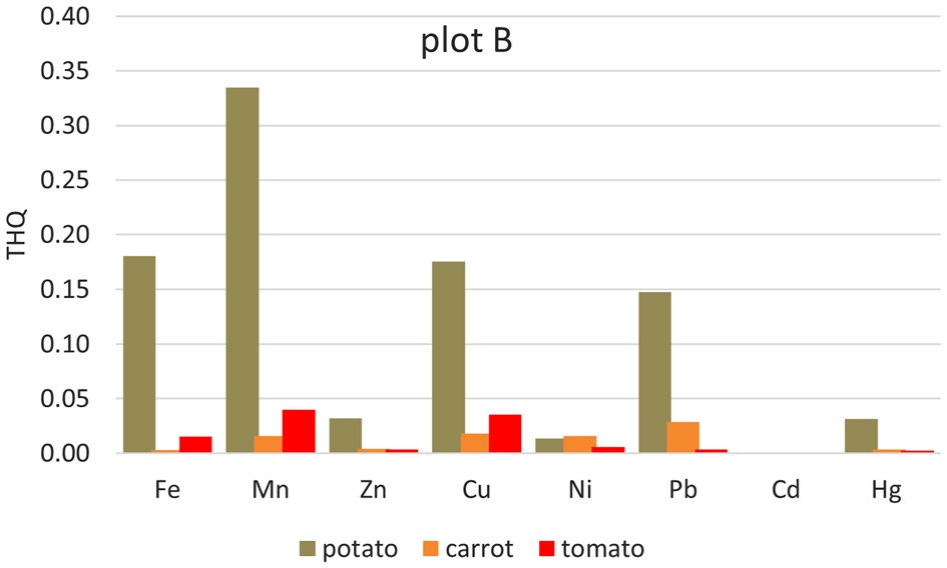
Target hazard quotient (THQ) of heavy metals in potatoes, carrots, and tomatoes grown in plot B.

**Figure 4:**
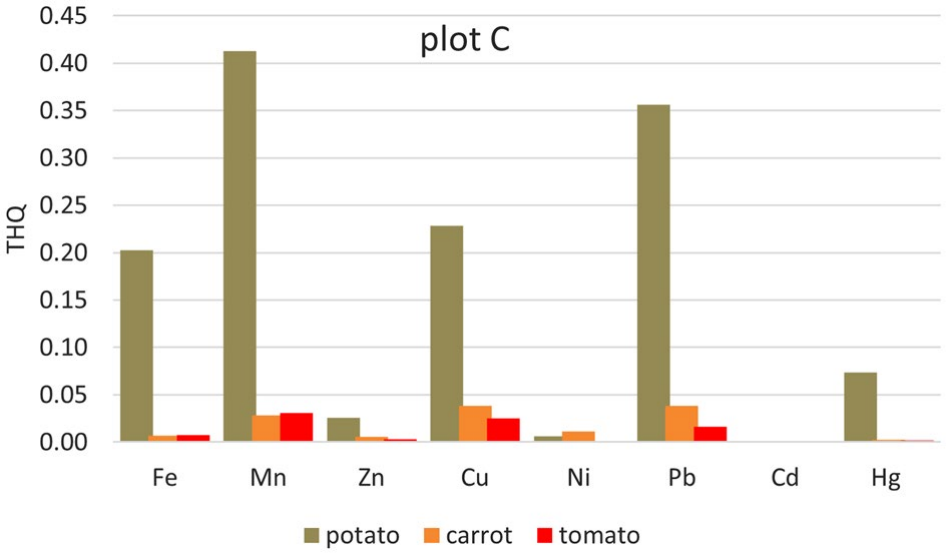
Target hazard quotient (THQ) of heavy metals in potatoes, carrots, and tomatoes grown in plot C.

**Figure 5:**
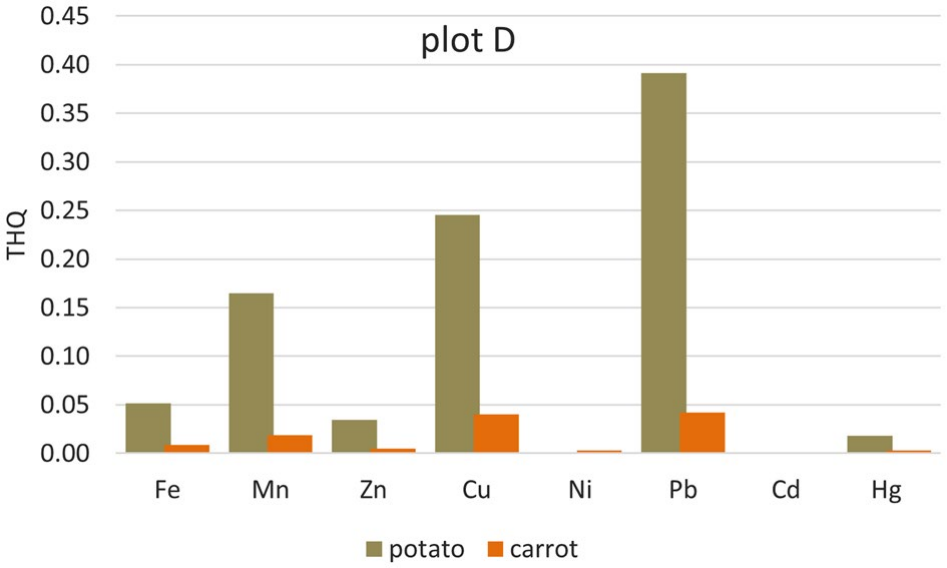
Target hazard quotient (THQ) of heavy metals in potatoes, carrots, and tomatoes grown in plot D.

**Figure 6:**
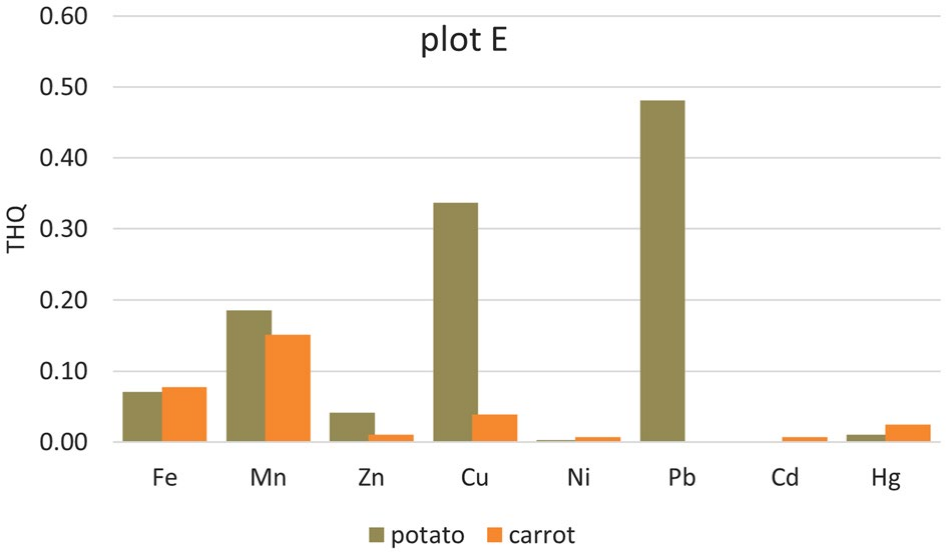
Target hazard quotient (THQ) of heavy metals in potatoes, carrots, and tomatoes grown in plot E.

Significantly higher THQ values were in potatoes from all five plots. Apart from Cd (where the Cd content was below the detection limit in the samples from all plots and Ni in the sample from plot A), the lowest values of Ni (from 3.521E−05 plot D to 0.01373 plot B) < Zn (0.0207 plot A–0.0417 plot E) < Fe (0.0518 plot D–0.2023 plot C, except plot B) (Figs. 2, 3, 4, 5 and 6). For other heavy metals, their order is ambiguous. The highest THQ values were for Pb (plot A: 0.3818, plot D: 0.3916, plot E: 0.4808), Mn (plot B: 0.3345), and Hg (plot C: 0.7363). Based on the results, it can be stated that the THQ of Ni, Zn, and Fe through vegetable consumption were much lower than THQ values of other metals. The THQ values of all analyzed metals were < 1.

The target hazard quotient was recognized as a useful parameter to assess the risk associated with the consumption of metal-contaminated foods^78^. THQ describes the non-carcinogenic health risk posed by exposure to the relevant toxic element. If THQ < 1, no non-carcinogenic health effects are expected. However, if THQ > 1, there is a possibility that adverse health effects may occur. THQ exceeding 1 statistically does not represent a probability of occurrence of adverse non-carcinogenic health effects^5,38,79^.

**Figure 7:**
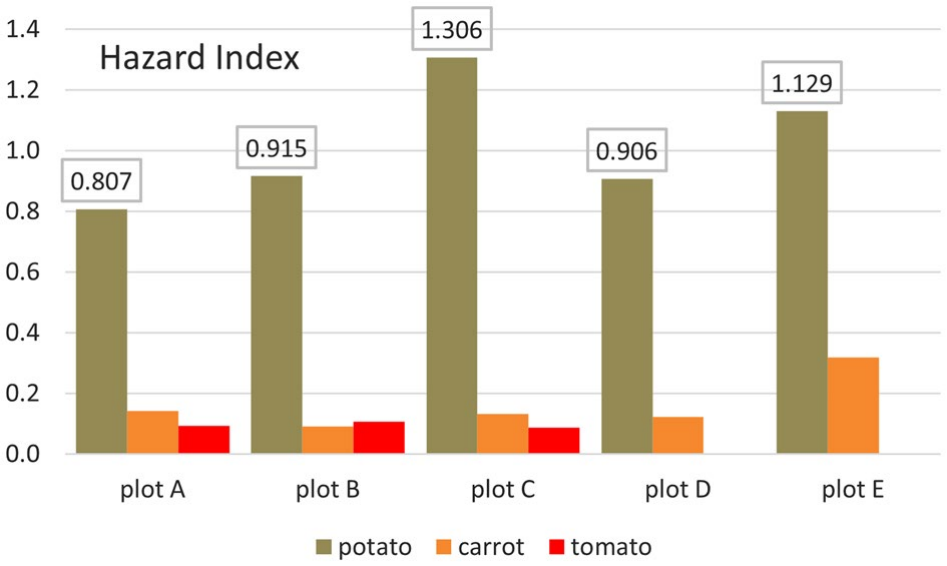
Hazard index (HI) of all heavy metals (Fe, Mn, Zn, Cu, Ni, Pb, and Hg) in potatoes, carrots, and tomatoes grown on individual plots.

Hazard index represents the cumulative effect of consuming several potentially hazardous elements. HIs for the eight heavy metals (Fe, Mn, Zn, Cu, Ni, Pb, and Hg) were evaluated in terms of the consumption of three vegetables (potato, carrot, and tomato). It is potatoes that can be characterized as a hazardous crop, as HI > 1 (for all plots) (Fig. 7). Other HIs ranged from 0.1001 (tomato, plot A) to 0.54.14 (carrot, plot E). Pb (25.6–39.3%), Hg (29.8–46.1%), and Mn (27.9–31.4%) contribute the most to the HI values.

HI anticipates that the consumption of a particular type of food would result in simultaneous exposure to several potentially toxic elements. Although the individual THQs for elements in food are less than one individual, the cumulative effect of consumption can have an adverse health effect. If HI > 1, there is a possibility of adverse non-carcinogenic effects on health^42^.

Increased intake of monitored heavy metals can have a negative impact on the health or even the life of the consumer. Environmental factors that may increase cancer risk in humans are identified in the monograph program, which is a key part of The International Agency for Research on Cancer (IARC). The IARC is a specialized agency for cancer of the World Health Organization (WHO). Metallic mercury and inorganic mercury compounds are not classifiable as to their carcinogenicity to humans (Group 3)^24^. IARC evaluated Ni (metallic)^80^ and Pb^81^ as possibly carcinogenic to humans (Group 2B), and Cd (cadmium compounds) as carcinogenic to humans (Group 1)^82^.

Presented results point to potential risks for the consumer resulting from soil contamination caused by old mining activities. For a more accurate evaluation, it would be suitable to extend the research by involving other crops, increasing the number of sampling points, and focusing on other elements (As, Mn, Se, etc.) that also may represent a risk for the consumer.

## Conclusion

One of the factors that adversely affect the environment is the negative impact of old environmental burdens. The Spiš region is one of the burdened areas in Slovakia due to the old, in many cases, already terminated mining activities. Concentrations of heavy metals in the soil have, in many cases, exceeded their maximum permissible levels in their pseudototal (Fe, Zn, Cu, Cd, and Hg) or bioavailable (Pb, Cd) form, compared to current legislation. The risk of soil contamination by monitored heavy metals is also confirmed by high values of soil pollution indices. All plots can be classified as very high contaminated with cadmium and mercury (based on C_f_), resp. all heavy metals (according to C_deg_). The riskiest element for all plots, according to I_geo_, is mercury. Based on the PLI, the pollution level can be characterized as moderately polluted to unpolluted. Increased levels of heavy metals in the soil were reflected in their accumulation in vegetables, especially potatoes. This crop also had the highest Estimated daily intake, Chronic daily intake, Target hazard quotient, and Hazard index. The obtained results showed the importance of soil and crop monitoring, especially in high-risk areas such as the Spiš region.

## Data Availability

All basic data supporting the results of this study are available from the corresponding author.
